# Systemic Curcumin-Human Serum Albumin in Proliferative Vitreoretinal Retinopathy: A Pilot Study

**DOI:** 10.7759/cureus.18645

**Published:** 2021-10-10

**Authors:** Annekatrin Rickmann, Andre Schulz, Bianca Bohrer, Maria Waizel, Lukas Bisorca-Gassendorf, Sami Al-Nawaiseh, Phillip Wakili, Kai Januschowski

**Affiliations:** 1 Ophthalmology, Eye Clinic, Knappschaft Hospital Saar, Sulzbach, DEU; 2 Research, Pharmbiotec GmbH, Saarbrücken, DEU; 3 Ophthalmology, University of Basel, Basel, CHE; 4 Ophthalmology, University Eye Hospital Tübingen, Tübingen, DEU

**Keywords:** retinal detachment, curcumin-hsa, curcumin, pvr, proliferative vitreoretinopathy

## Abstract

Objectives

The purpose of this study is to compare the risks of novel postoperative curcumin infusion in patients with increased proliferative vitreoretinal retinopathy (PVR) after retinal detachment with steroid infusion or no treatment.

Methods

This was a prospective, non-randomized pilot study of 15 eyes of 15 patients (mean age 68 ± 7 years) with retinal detachment, macula off, and flare >15 pc/ms. Postoperatively, the patients received either curcumin-HSA (human serum albumin) infusion (C, n=5), prednisolone infusion (P, n=5), or no therapy (N, n=5) for three days. The outcome measures included postoperative PVR rate, the number of vitreoretinal surgeries (VRS) required, epiretinal membrane development, and visual acuity (VA).

Results

All patients had a preoperative VA of hand movements, macula-off detachment situation, and two quadrants rhegmatogenous retinal detachment. Patients underwent VRS at a mean time of 5.6 ± 1.5 (C), 4.9 ± 2.0 (P), 4.7 ± 1.2 (N) days after first recognized symptoms. Postoperative PVR developed just in one eye (P) after 16 days and required VRS due to PVR retinal detachment. The remaining 14 patients of group C and N did not develop PVR. BCVA improved six months post surgery to 0.56 ± 0.31 (P), 0.53 ± 0.19 (D), 0.53 ± 0.17 (N) logMAR. There were no side effects nor complications related to the postoperative infusions.

Conclusions

In this pilot study, we demonstrated that a postoperative application of curcumin infusion is a safe option in patients with an increased risk of PVR. Whether or not PVR can be reduced by curcumin infusion would require to be investigated in larger, randomized clinical trials.

## Introduction

Proliferative vitreoretinal retinopathy (PVR) is considered to be the main cause of failure after retinal detachment (RD) surgery [1]. PVR is a complex vitreoretinal wound healing process that occurs in a sequence of three overlapping phases: inflammation, cell migration and proliferation, and remodeling of the retina and extracellular matrix [2].

Over the last decades, vitreoretinal surgical techniques have evolved but prevention of PVR still depends on the success of the primary RD surgery. Nevertheless, the postoperative incidence of PVR in prospective studies is between 4% and 34% [3,4]. Therefore, it appears that there is a need for concomitant pharmacological treatment that could prevent or halt the progression of PVR [5].

Despite numerous advances, the pathogenesis of PVR is still not completely understood. The development of molecular biology techniques has led to the elucidation of many different cellular and biological aspects of PVR [4], but at this time there is no proven pharmacologic agent for the treatment or prevention of PVR.

Curcumin may be a promising approach as it targets many sequences of molecular and cellular signaling pathways (i.e. miRs, signal transducers, and activators of transcription 3, p-AKT, PVR, and Rb) involved in the pathogenesis of retinal diseases [6]. Curcumin is a polyphenol extracted from turmeric that has long been used to treat a variety of diseases, including neurodegenerative and inflammatory disorders. It controls inflammation, cell growth, and apoptosis and is therefore suitable for the prevention and treatment of certain diseases due to its antioxidant and anti-inflammatory properties and excellent safety profile [7]. An experimental PVR model has demonstrated that curcumin could have great potential as a therapeutic agent by inhibiting various inflammatory factors [8]. However, the clinical application of curcumin has been limited by the poor solubility and low bioavailability of this molecule [9]. Addressing this, we used a curcumin formula with HSA (human serum albumin) which has an improved bioavailability.

The aim of this pilot study was to evaluate the applicability of this curcumin infusion in clinical routines in patients with an increased risk of PVR after retinal detachment compared to steroid infusion or no postoperative treatment.

## Materials and methods

This paper details a prospective, non-randomized, single-center, consecutive pilot study on 15 patients (15 eyes, mean age 68 ± 7 years) with a primary rhegmatogenous retinal detachment in the Eye Clinic, Suzbach/Saar, Germany.

Included were patients with primary rhegmatogenous retinal detachment (< 2 weeks) with an indication for pars plana vitrectomy (ppV) without combined cataract surgery with a laser flare value >15 pc/ms (photon counts/ms) (Laser Flare Photometer: Kowa FM-700, Kowa Optimed Deutschland GmbH, Düsseldorf, Germany). We decided on this value because it could be shown that the risk of developing PVR was 16 times higher with a laser flare value >15 pc/ms [10].

Exclusion criteria were longer existing (or unclear since when) retinal detachment, history of trauma, giant tears, visible PVR grade B/C (according to [11]), pre-existing diseases (retinal dystrophy, chronic inflammatory diseases, retinal vascular diseases, diabetic retinopathy, uveitis, glaucoma, condition after intraocular surgery (except uncomplicated phacoemuslification more than three months ago), condition after laser retinopexy/exo-cryopexy), aphakia, vitreous hemorrhage, as well as systemic diseases or drugs (e.g. chemo-/immunotherapy) which could have an influence.

After clarification of the study content, patients could decide for themselves whether they wanted to receive systemic therapy with Solu-Decortin H 250mg infusion (186.7mg prednisolon) (Merck KGaA, Darmstadt, Germany) (group P) or systemic 300mg curcumin-HSA infusion (Viktoria Apotheke, Saarbrücken, Germany) (group C) or no systemic therapy (group N). We initially limited participation in the study to five persons in each group. Possible side effects of curcumin include gastrointestinal complaints or yellowing of the injection site.

**Figure 1:**
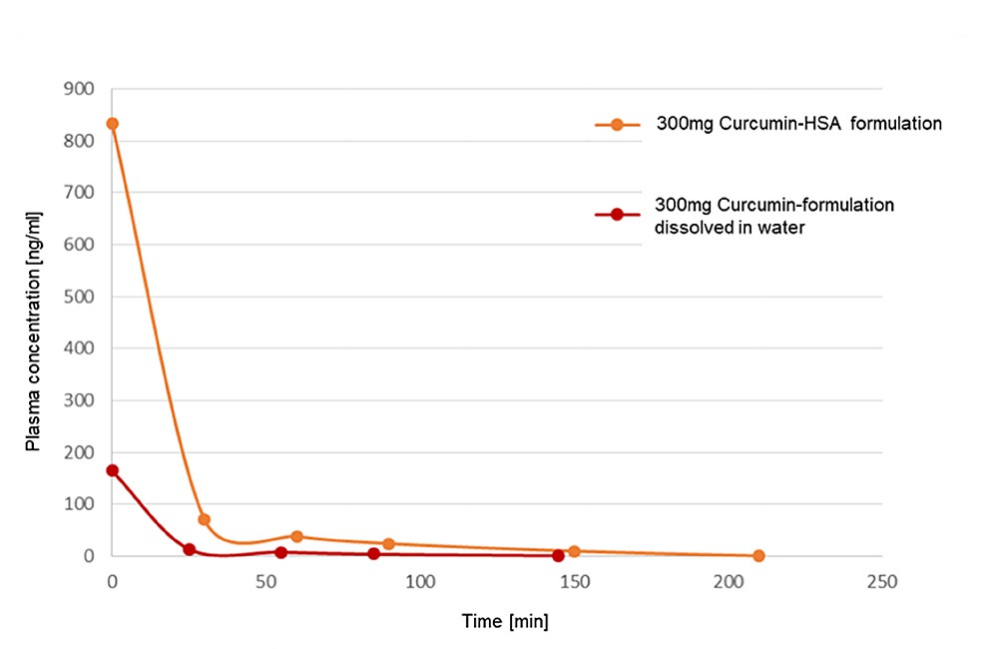
Comparison of the plasma concentration Comparison of the plasma concentration after infusion of curcumin-HSA formulation with 300mg curcumin compared to the same dosage of a formulation dissolved in water. It shows a significantly prolonged plasma presence of free, non-metabolized curcumin in the HSA-bound curcumin formulation compared to the water-dissolved curcumin formulation. The presence of free and thus therapeutically effective curcumin is extended to 210 minutes after infusion in the HSA-bound formulation. The initial plasma concentration after the 120-minute infusion of the HSA formulation shows a five-fold higher amount of curcumin in the blood than with the water-dissolved curcumin variant.

We used a curcumin formula with HSA (human serum albumin) (curcumin-HSA, Viktoria Apotheke, Saarbrücken, Germany), which improves the bioavailability. We were already able to show that the plasma concentration after infusion of the used curcumin-HSA formulation with 300mg curcumin compared to the same dosage of a formulation dissolved in water shows a significantly prolonged plasma presence of free, non-metabolized curcumin in the HSA-bound curcumin formulation compared to the water-dissolved curcumin formulation (Figure 1).

Currently, this curcumin-HSA infusion is used regularly in patients undergoing chemotherapy to alleviate the side effects.

In PVR, cell proliferation reaches its peak in the first four days after RD and decreases by Day 7 [12]. Therefore, we decided to perform the systemic therapy over three days. After written patient consent, the groups P and C received one infusion each for three days postoperatively, starting directly after surgery in the operation room.

PVR is a relatively acute process, with 95% of cases occurring within the first 45 days [13]. Therefore, patients were scheduled for two weeks, four to six weeks, three and six months. Preoperatively and during all visits an OCT (optical coherence tomography; Heidelberg Engineering, Germany) and fundus examination was performed independently by two retina specialists to check the PVR status. We defined PVR according to the classification revised by the Retina Society [11]. The primary outcome measure was the rate of postoperative PVR and the number of additional required vitreoretinal surgeries (VRS). The second outcome measure was best corrected visual acuity (BCVA).

This study was performed according to the tenets of the Declaration of Helsinki and was approved by the local ethics committee (Ärztekammer Saarland, 243/14). Written informed consent was obtained from the patients after an explanation of the nature and possible consequences of the study.

The ppV was performed under a standard ophthalmic operating microscope (Lumera 700 microscope, Carl Zeiss Meditec Inc., Germany) by only one experienced surgeon. A standard 23-gauge suture-less vitrectomy system was used (Eva, D.O.R.C., Zuidland, Netherlands) with the endoillumination set to 80% and a two-dimensional cutter (TDC Cutter 23G D.O.R.C., Zuidland, Netherlands) set to 8000 cpm during core vitrectomy (maximum vacuum 450 mmHg) peripheral vitrectomy (maximum vacuum 250 mmHg) and shaving for all procedures. All patients had a posterior vitreous detachment. A core vitrectomy was performed followed by a reattachment of the retina using ultrapure perfluorocarbons (F-Octane 1.76 g/cm3 C8F18, Geuder GmbH, Heidelberg, Germany) under visual control. Thereafter an external 360° indentation and shaving of the vitreous base was performed using an indentor and direct visualization with an endoillumination with the operating microscope and a handheld lens (OLV2 Ocular Landers HRI Vitrectomy Lens). At the end of surgery, C2F6 tamponade was used in all cases. Finally, the infusion cannula was removed and the sclerotomies were sutured if necessary (Vicryl 7-0, Johnson & Johnson Intl., New Brunswick, NJ). The patients were instructed to adhere to a position that would allow for the ideal effectiveness of the tamponade vector.

Statistical analysis was performed using SPSS Statistics v.22 (IBM Corp., Armonk, NY). All results are presented as mean and standard deviation (± SD). Kruskal-Wallis test was used to test for differences between groups. A p-value of <0.05 was defined as statistically significant.

## Results

The patient’s characteristics are shown in Table 1.

**Table 1:** Patients characteristics VA – Visual acuity IOP – intraocular pressure *after 16 days

	Group P (prednisolone) n=5	Group C (curcumin) n=5	Group N (No therapy) n=5	p-value
PREOPERATIVE				
Ratio of male/female	4/1	3/2	4/1	0.31
Patient age in years	64.9±3	70.1±5	69.4±9	0.74
Preoperative flare in pc/ms	18.7±2.2	22.8±5.6	19.2±1.4	0.52
Preoperative IOP	19±1.5	15±1.2	15±2.2	0.14
Number of days of symptoms until surgery	4.9±2.0	5.6±1.5	4.7±1.2	0.75
Preoperative phak/pseudophak	2/3	¼	1/4	0.2
Number of foramina	2.4±1.8	2.1±0.9	2.3±1.7	0.76
POSTOPERATIVE				
Postoperative PVR development	1/5*	0/5	0/5	0.27
Flare in pc/ms, two weeks	5.1±2.7	5.2±1.3	5.0±1.8	0.82
VA in logMAR, six months	0.56±0.31	0.53±0.19	0.53±0.17	0.44

No statistically significant difference was found between the groups preoperatively. Intraoperatively up to five retinal breaks were present and predominantly superior (Primary hole position: superior/inferior=2/1 in all groups). All patients had a retinal detachment involving two quadrants and a macula-off detachment situation with a mean total time of 5±1.9 days since the onset of symptoms and surgical treatment. All patients demonstrated visual acuity of hand movements preoperatively. Patients who received a curcumin-HSA infusion were asked daily about their tolerability. All patients did not report any abnormalities at any time and thus tolerated the infusion well. Likewise, all patients on Solu-Decortin H infusion also reported no abnormalities.

One patient of group P developed a retinal detachment again 16 days after initial treatment with an inferiorly located PVR membrane (PVR B), which was operated on immediately. Prior to initial treatment, a one-break situation was present at the 1 o'clock position with retinal detachment of the superior hemisphere. The remaining 14 patients did not develop PVR over six months. Also, no macular epiretinal membrane developed during this period, and no further surgery was necessary for these 14 patients. The postoperative visual acuity in the three groups is also not statistically significant to each other (Table 1).

## Discussion

In this pilot study, we were able to show that the application of a curcumin-HSA infusion is possible with good tolerability and that no PVR occurred within a period of six months.

The promising properties of curcumin give rise to hope for a supportive preventive therapy option since there is currently no preventive treatment other than the important adequate primary surgical performance. One of the main reasons for this is that the pathogenesis of PVR has not yet been fully understood but is considered multifactorial [5].

Various factors are considered to be particularly important in various PVR models [14-17], but the question arises as to the role of these experimental PVR models in a not fully understood multifactorial pathogenesis [5]. And indeed, there is a need for an approach that does not deal with a single factor but can influence the imbalance in several ways. Curcumin would therefore be a promising approach as it has several promising effects on PVR prevention and treatment [18]. It reverses epithelial-to-mesenchymal transition (EMT) [19], inhibits RPE cell proliferation [20], promotes human RPE cell death through organized apoptotic pathways [21], and induces cell cycle arrest by activating multiple checkpoints [22]. Thus, pathological cell growth triggered by growth factors through stress and inflammation can be suppressed.

Although inflammation plays an important role in PVR formation, it is not the only factor. Also, neuroprotection could play an additional role in preventing PVR [5]. Curcumin could also play a role here, as several preclinical and clinical studies assessed the therapeutic effects of curcumin on retinal disorders, such as glaucoma, diabetic retinopathy and age-related macular degeneration [23,24].

These experimental studies encourage that curcumin could be used for the prevention of PVR, but so far, clinical application is limited yet. Curcumin has indeed a high level of safety and low toxicity [22], but the biomedical potential of curcumin is not easy to use, given its low solubility and oral bioavailability and possible lack of purity of the extracts [9]. In our study, we used a novel, GMP-produced curcumin formulation with an improved bioavailability using HSA. The patients in this study who received curcumin did not complain of any intolerances.

Based on the hypothesis that PVR pathogenesis is due to inflammation, our surgeons used Solu-Decortin H 250mg infusion regularly over three days due to the lack of alternatives in patients who might have a PVR risk. Therefore, we decided to include this as a comparison group. However, human studies failed to demonstrate a beneficial effect of steroids [25]. Even so, in the group with systemic steroid therapy, one patient had PVR after 16 days. However, if all patients of the study are considered, this corresponds with 1/15 patients (7%) to the statistical incidence described in the literature [3,4]. Of course, several risk factors as well as iatrogenic complications are important factors for the development of postoperative PVR [26]. However, preoperative risk factors were shown to be comparable in our three groups.

It is however of enormous importance to avoid such a PVR because the anatomic success rate has been reported to be 45-85% [27,28] and the final functional success rates of PVR detachment surgery are just 26-67% (defined as a final visual acuity of 5/200 or better) [27,28]. The best prevention of PVR is of course still the success of the initial surgery [29]. Naturally, the relative importance of the different factors in the formation and progression of PVR needs further investigation in order to develop more efficient (pharmacological) therapeutic approaches [29].

Limitations of the study are the small sample size and missing randomization. Therefore, a definitive statement on the possible clinical prevalence of PVR cannot be made. 

## Conclusions

In conclusion, a definitive statement on the possible clinical prevalence of PVR cannot be made. However, curcumin, due to its possible multifactorial mechanism of action, might be a potential additive therapy option in the prevention of PVR. But randomized clinical trials are needed.
